# A comparison of the neuromuscular, cardiopulmonary and muscle oxygenation responses to single‐ and double‐leg cycling in older adults

**DOI:** 10.1113/EP093678

**Published:** 2026-05-30

**Authors:** Emily Dodd, Callum G. Brownstein

**Affiliations:** ^1^ School of Biomedical, Nutritional, and Sport Sciences, Faculty of Medical Sciences Newcastle University Newcastle upon Tyne UK

**Keywords:** cycling, exercise, fatigability, oxygen transport

## Abstract

Previous work in younger adults has shown that during small muscle mass exercise, the tolerable degree of neuromuscular impairment at task failure is greater than during large muscle mass exercise. However, no study has compared the neuromuscular responses to small and large muscle mass exercise whilst matching exercise modality in older individuals. This study compared neuromuscular, cardiopulmonary and muscle oxygenation responses between incremental single‐ and double‐leg cycling (SLC and DLC). Eleven older adults (68 ± 4 years) completed incremental cycling with 3 min stages, with SLC work rate set at half of DLC. Between each stage, neuromuscular function of the right knee extensor was assessed using a customised recumbent cycle ergometer, with measurements including maximal voluntary contraction (MVC), voluntary activation (VA) and quadriceps potentiated twitch force (*Q*_tw, pot_). Oxygen consumption (${\dot{V}}_{O_{2}}$), minute ventilation (${\dot{V}}_{E}$), heart rate (HR), and near‐infrared spectroscopy (NIRS) and vastus lateralis electromyography (EMG) were measured throughout. Time to task failure did not differ between SLC and DLC (*P *= 0.616). However, reductions in *Q*_tw,pot_ were greater at task failure following SLC (45.7% ± 18.8%) than DLC (30.0% ± 14.0%; *P *= 0.049), with no differences in MVC (*P *= 0.219) or VA (*P *= 0.547). Electromyography measured during cycling was 22.5 ± 23.0% higher at task failure during SLC than DLC (*P *= 0.001). Pulmonary ${\dot{V}}_{O_{2}}$, ${\dot{V}}_{E}$ and HR were lower throughout SLC than DLC (*P *≤ 0.010), while oxygenated haemoglobin was 8.8 ± 8.0% higher throughout SLC than DLC (*P *= 0.006). These results demonstrate that small muscle mass exercise permits a greater tolerable degree of contractile impairment to be incurred, with a reduced cardiopulmonary demand, relative to large muscle mass exercise in older adults.

## Abstract (highlights)

- **What is the central question of this study?**
  This study assessed the neuromuscular, cardiopulmonary and muscle oxygenation responses to incremental single‐ and double‐leg cycling in older adults.
- **What is the main finding and its importance?**
  Impairments in contractile function at task failure were higher following single‐ than double‐leg cycling, alongside higher muscle electromyography. Concurrently, the cardiopulmonary responses were attenuated during single‐leg cycling, while oxygenated haemoglobin was higher. These results demonstrate that a higher degree of contractile impairment can be tolerated during small versus large muscle mass exercise in older adults.

## 1 INTRODUCTION

Neuromuscular fatigability is defined as an exercise‐induced reduction in the capacity of muscle to generate force or power, and is determined by two general mechanisms, including impairments in nervous system voluntary activation (VA) of muscle and reductions in contractile function (Brownstein et al., 2021; Gandevia, 2001). During high‐intensity exercise involving locomotor muscle groups, the amount of active muscle mass recruited during exercise has been shown to strongly influence the magnitude of neuromuscular fatigability at task failure (Rossman et al., 2012; Weavil et al., 2018; Zhang et al., 2021, 2024). Specifically, studies that have manipulated active muscle mass during exercise performed to task failure have consistently shown that neuromuscular fatigability – measured through reductions in maximal voluntary contraction (MVC) force and evoked resting twitch responses, indicative of impaired contractile function – are significantly greater following small muscle mass exercise (e.g., single‐leg cycling or knee‐extension exercises) compared with large muscle mass exercise (e.g., double‐leg cycling), with negligible differences in VA (for meta‐analysis, see Zhang et al., 2025). These findings suggest that the magnitude of impairments in neuromuscular function is modulated by the size of the muscle mass involved, with smaller muscle mass exercise eliciting more pronounced neuromuscular impairments at task failure due to mechanisms residing within the muscle.

The greater magnitude of neuromuscular fatigability during small muscle mass exercise likely relates, at least in part, to the ability to reach a higher degree of metabolic perturbation prior to task failure as a result of attenuated neural and cardiopulmonary constraints (Thomas et al., 2018). Specifically, during large muscle mass exercise, limitations in cardiac output and pulmonary constraints associated with high levels of ventilation limit oxygen delivery to the working muscles (Dominelli et al., 2021). These systemic demands are also associated with higher perceptions of effort and dyspnoea during high‐intensity large versus small muscle mass exercise (Abbiss et al., 2011; Zhang et al., 2021). Collectively, these factors are thought to constrain exercise tolerance and the magnitude of muscle metabolic perturbation incurred. In contrast, these central constraints are markedly reduced during small muscle mass exercise, permitting a greater muscle activation, metabolic disturbance and impairment in contractile function to be tolerated before task failure occurs (Rossman et al., 2012; Zhang et al., 2021). Indeed, concurrent with the greater reduction in resting twitch responses, studies have also demonstrated higher electromyographic (EMG) activity at peak exercise during small compared with large muscle mass exercise (Rossman et al., 2012; Zhang et al., 2021). Given that the metabolic perturbations associated with high‐intensity exercise are thought to represent key initiators of transcriptional factors that promote peripheral adaptations, small muscle mass exercise has been suggested as an effective training modality for enhancing peripheral vascular and metabolic function whilst mitigating the central constraints associated with large muscle mass exercise (Heidorn et al., 2023). Indeed, several studies support the efficacy of small muscle mass exercise training in promoting peripheral adaptations that translate to improvements in exercise tolerance during whole‐body exercise (Dolmage & Goldstein, 2008; Gordon et al., 2019; Tyni‐Lenné et al., 1999; for review, see Brownstein, 2026).

While numerous studies have demonstrated greater neuromuscular fatigability at task failure following small compared with large muscle mass exercise, this work has predominantly been conducted in young, healthy individuals, with relatively little attention given to older adults (Rossman et al., 2012; Zhang et al., 2021, 2024). Investigating the influence of active muscle mass on fatigability and exercise tolerance is of relevance in older individuals given the cardiopulmonary limitations associated with older age. For example, older adults exhibit lower maximum cardiac output and an increased work of breathing and dyspnoea due to impairments in respiratory compliance (Ferrari et al., 2003; Smith et al., 2018; Weavil et al., 2022). These central constraints could attenuate the degree of metabolic stress incurred during high‐intensity large muscle mass exercise. This is of particular relevance given that older age is associated with impairments in peripheral vascular and metabolic function (Carter et al., 2015; Landers‐Ramos & Prior, 2018). Accordingly, determining whether manipulation of active muscle mass modulates neuromuscular fatigability in older adults is important for understanding both the mechanisms limiting exercise tolerance and the potential utility of small muscle mass exercise as a training strategy in this population.

To date, only one study has compared fatigability between small and large muscle mass exercise in older adults. Specifically, Weavil et al. (2018) found greater impairments in MVC and resting evoked twitch responses following dynamic single‐leg knee extension compared with cycling. However, a potential confounding factor in these findings is the substantial restriction of blood flow that can occur due to the sustained muscular tension inherent to dynamic knee extension exercise. A more appropriate model to compare fatigability between small and large muscle mass exercise without altering muscle contraction patterns is using counterweight single‐leg and double‐leg cycling. Indeed, previous work has shown that relative knee extensor work is comparable between single‐ and double‐leg cycling (SLC and DLC) at matched relative power outputs when using a counterweight system (Elmer et al., 2016). Accordingly, the primary aim of the present study was to compare neuromuscular fatigability in response to SLC and DLC in older adults. Given the heightened cardiopulmonary constraints associated with ageing, which may limit whole‐body exercise tolerance and restrict the degree of metabolic perturbation and contractile impairment that can be attained, it was important to determine whether reducing the active muscle mass permits a greater magnitude of fatigability to develop in this population. We hypothesised that neuromuscular fatigability would be exacerbated at task failure during single‐ compared with double‐leg cycling, concurrent with reduced cardiopulmonary demands, indicating that manipulation of active muscle mass remains an effective means of increasing peripheral metabolic stress in older adults.

## 2 METHODS

### 2.1 Ethical approval

All participants provided written informed consent prior to commencing testing. The study received ethical approval from the Newcastle University Faculty of Medical Sciences Research Ethics Committee (2786/45659) in accordance with the ethical standards established in the *Declaration of Helsinki*, apart from registration in a database.

### 2.2 Participants

Eleven older adults (age 68 ± 4 years, stature 174.9 ± 7.5 cm, mass 72.1 ± 10.8 kg), including two females, provided written informed consent for the study. Participants were asked to refrain from strenuous exercise and alcohol consumption in the 24 h prior to each visit, and to arrive at the laboratory 2–3 h post‐prandial. Inclusion criteria included being aged ≥60 years. Exclusion criteria included any cardiorespiratory, neurological or metabolic health disorders, any musculoskeletal injuries, and any contraindications to receiving peripheral nerve stimulation.

### 2.3 Design

Participants visited the laboratory on three occasions. Visit 1 comprised a familiarisation with the study procedures, including assessments of neuromuscular function and with performing SLC. Visits 2 and 3 were randomised in order, and included step‐incremental exercise performed to task failure during either DLC or SLC. Each step was 3 min in duration, with neuromuscular function measured at baseline, between each step, and at task failure. Throughout the incremental tests, gas exchange, ventilation, vastus lateralis (VL) muscle oxygenation (using near‐infrared spectroscopy [NIRS]) and electrical activity (using electromyography [EMG]) were measured. Visits 2 and 3 were separated by a minimum of 1 week.

### 2.4 Experimental protocol

#### 2.4.1 Incremental cycling test

**FIGURE 1:**
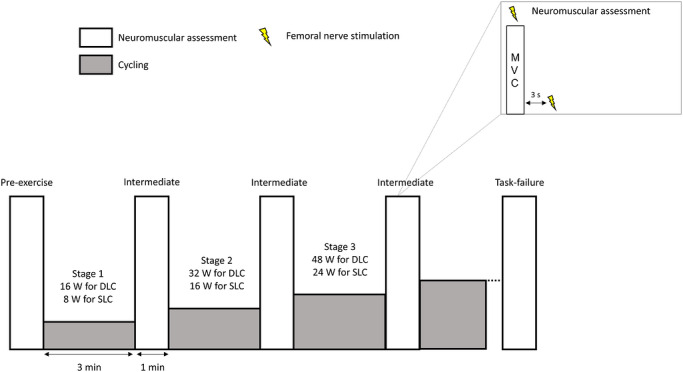
Illustration of incremental cycling test protocol for double‐leg cycling (DLC) and single‐leg cycling (SLC). Stage increments for DLC were 16 W per stage up to stage 5, and 26 W thereafter. Power outputs at each stage were halved for SLC. The neuromuscular assessments performed pre‐exercise, between stages and at task failure consisted of a maximal voluntary contraction (MVC) of the right knee extensor with superimposed and post‐MVC femoral nerve stimulation.

Both SLC and DLC incremental exercise tests were performed on an electromagnetically braked recumbent cycle ergometer (Lode Corival Recumbent CPET, Lode, Groningen, the Netherlands). SLC was performed with the right leg, with a 10 kg counterweight placed on the left pedal (Bini et al., 2015; Elmer et al., 2016). A graphical representing of the incremental cycling test is displayed in Figure 1. Participants remained quietly seated on the cycle ergometer for a 5 min resting period prior to commencing cycling. DLC commenced at 16 W, with 16 W increments each stage up until stage 5 (i.e., 80 W). Beyond stage 5, the increments increased to 26 W until task failure was reached. For SLC, increments were set at half that of DLC, i.e., 8 W for the first five stages and 13 W thereafter. This progression, employing smaller increments initially followed by larger increments after five stages, was designed to ensure sufficient stages were completed to permit between‐modality comparisons at equivalent relative time points, while preventing excessively long test durations in fitter participants. The use of 3 min stages is consistent with step‐incremental protocols designed to assess neuromuscular fatigability (Brownstein et al., 2022; Doyle‐Baker et al., 2018), as this duration permits sufficient development of metabolic and cardiorespiratory responses to the imposed work rate prior to neuromuscular assessment. Each stage was interspersed with a 1 min interval, during which neuromuscular function was assessed (see ‘Neuromuscular function assessment’ below). Following completion of the neuromuscular assessment, participants performed unloaded cycling for the remainder of the 1 min interval, before power output was increased to the target level. Participants were instructed to maintain a cadence of 60–90 rpm throughout the test. The test was terminated once the participant's self‐selected cadence decreased by 10 rpm, or they voluntarily disengaged from the task despite strong verbal encouragement, whichever came sooner.

#### 2.4.2 Neuromuscular function assessments

Neuromuscular assessments were performed on the right knee extensor. The baseline assessment began with the determination of the appropriate intensity for electrical nerve stimulation (described under ‘Femoral nerve stimulation’ below). Subsequently, an isometric warm‐up consisting of submaximal contractions at 25%, 50% and 75% of perceived maximum force, separated by 10 s, was performed. Next, participants performed 2 × 3 s isometric MVCs without stimulation, followed by three MVCs with superimposed supramaximal electrical stimulation to the femoral nerve, and the same electrical stimulation delivered at rest 3 s following the MVC to evoke a potentiated twitch (quadriceps potentiated twitch force, *Q*_tw, pot_). All baseline MVCs were separated by 1 min. During the neuromuscular assessments between stages and at task failure, one MVC was performed, with a stimulation delivered during and 3 s following the MVC

### 2.5 Instrumentation

#### 2.5.1 Recumbent cycle ergometer

**FIGURE 2:**
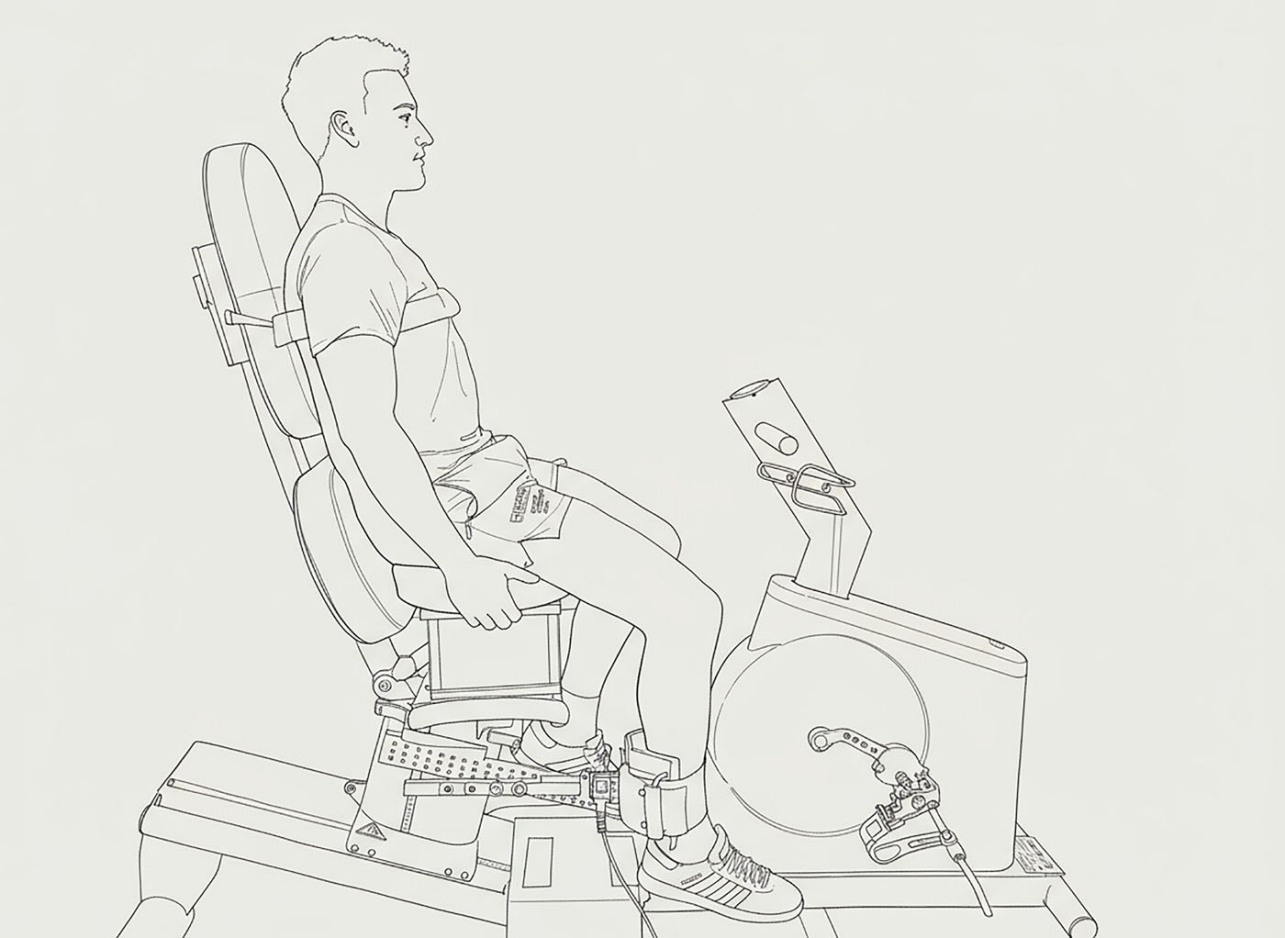
Recumbent cycle ergometer used for cycling bouts and to assess neuromuscular function. The ergometer was modified to include an adjustable rod to the base of the seat, which extended to a load cell situated immediately behind a non‐compliant cuff attached above the ankle malleoli. The seat was raised by fixing a box beneath the seat to ensure that the foot did not contact the ground during neuromuscular assessments.

The present study used a modified recumbent cycle ergometer to permit the rapid (<15 s) assessment of neuromuscular function between stages and at task failure (Figure 2). The ergometer was modified by fixing an adjustable rod to the base of the ergometer seat, immediately underneath the seat. The height and length of the rod could be adjusted to align with the participants’ right leg, 2 cm superior to the ankle malleoli. The rod extended towards this position, and a load cell was situated immediately behind the participants’ right ankle. The load cell was attached to the participants’ right leg using a non‐compliant cuff. In addition, to ensure that the participants’ foot did not make contact with the floor during neuromuscular assessments, the seat of the ergometer was raised by fixing a box underneath the seat (Figure 2). During neuromuscular assessments, the position of the left foot was standardised by resting the foot against a wooden block on the left side of the base of the ergometer. This configuration ensured consistent posture and limb positioning across neuromuscular assessments for both DLC and SLC. Immediately prior to neuromuscular assessments, participants removed their feet (for DLC) or right foot (for SLC), and the researcher immediately strapped the right leg to the cuff before subsequently commencing the neuromuscular assessment. Participants were attached to the bike chair using a belt secured around the chest, helping to minimise movement of the torso during contractions. The hip and knee angles were 60° flexion. The position of the chair and of the adjustable rod was determined during the familiarisation visit and kept consistent for Visits 2 and 3.

#### 2.5.2 Force and electromyography

Force (N) was measured using a calibrated load cell (S‐Beam Load Cell, Force Logic, London, UK), with the force signal transmitted to a PowerLab system (16/35; ADInstruments, Bella Vista, Australia) via a load cell amplifier (A50 Load Cell Amplifier, Force Logic). EMG activity of the VL was measured throughout cycling using wireless electrodes (Pico EMG, Cometa, Bareggio, Italy). Self‐adhesive surface electrodes (10 mm recording diameter; MediTrace 100, Covidien, Mansfield, MA, USA) were placed on the belly of the right VL using a bipolar configuration with a 30 mm interelectrode distance. The distal edge of the EMG electrode pair was positioned 5 cm proximal to the distal border of the VL, as identified through palpation. Prior to electrode placement, the skin was shaved, abraded and cleaned with isopropyl alcohol to limit impedance. The EMG signals were amplified, bandpass filtered (10–500 Hz) and analog‐to‐digitally converted at a sampling rate of 2 kHz by a wireless receiver (Wave Plus Receiver, Cometa). The receiver was connected to a BNC patch panel (Cometa), which transmitted the EMG signal to the PowerLab system.

### 2.6 Femoral nerve stimulation

Stimulations of the right femoral nerve were delivered via a constant‐current stimulator (DS7R; Digitimer, Welwyn Garden City, UK) using single rectangular electrical pulses (1 ms duration) and a 400 V maximal output voltage. The cathode electrode (10 mm stimulating diameter; Meditrace 100, Covidien) was placed on the femoral triangle and a 50 × 100 mm anode (Easysnap; Compex, Guildford, UK) placed on the gluteal fold. Electrical stimuli were first administered at 30 mA and were then increased in 30 mA increments until the maximum twitch amplitude and maximal compound muscle action potential (*M*_max_) were elicited. The resulting stimulation intensity was then increased by 30% to account for activity‐induced changes in axonal excitability (187 ± 61 mV).

#### 2.6.1 Gas exchange, ventilation and heart rate

Gas exchange and ventilation were measured throughout the DLC and SLC visits. Before each visit, the metabolic cart (Metalyzer 3B, Cortex Medical, Leipzig, Germany) was calibrated following the manufacturer's guidelines to gases of known concentrations (O_2_, 15.15%; CO_2_, 5.03%). Ventilatory volumes were calibrated using a 3 L syringe. Heart rate (HR) was measured using a 12‐lead ECG (Custo Cardio 100 BT, Custo Med GmbH, Ottobrunn, Germany).

#### 2.6.2 Near‐infrared spectroscopy

Changes in tissue oxygen saturation (TSI), total haemoglobin (THb), oxygenated haemoglobin (O_2_Hb) and deoxygenated haemoglobin (HHb) of the right VL were measured using dual wavelength (760 and 850 nm), continuous‐wave near‐infrared spectroscopy (NIRS; PortaMon, Artinis Medical Systems, Elst, Netherlands), sampled at 10 Hz. The source–detector distances of the NIRS probe were 30, 35 and 40 mm. The NIRS probe was placed immediately proximal to the EMG electrodes, and was secured to the skin with adhesive tape and tensor bandage to avoid motion and ambient light influences. Prior to exercise during Visits 2 and 3, a physiological calibration was performed. A 12 cm pneumatic cuff (SC12D, Hokanson, Bellevue, WA, USA) was placed proximally to the NIRS probe and attached to an electronically controlled rapid cuff‐inflator (E20, Hokanson), supplied with air using an air compressor (Bambi BB15V, Bambi Air Ltd, Birmingham, UK). The cuff was inflated to 300 mmHg for 5 min while the participants lay supine, after which a plateau in NIRS signals was obtained in all participants. Upon deflation of the cuff, the participants remained still for a further 3 min to capture the post‐occlusive reperfusion response and to identify the extrema in the NIRS‐derived signals. A plateau in NIRS signals was observed by 3 min in all participants.

### 2.7 Data analysis

#### 2.7.1 Time normalisation and selection of analysis time points

Time to task failure (TTF) was defined as the total duration from the onset of stage 1 until task failure. To examine responses across the exercise bout independent of between‐condition differences in TTF, values were expressed at 25%, 50%, 75% and 100% of individual TTF. For the 25%, 50% and 75% time points, the corresponding time was identified from each participant's TTF and assigned to the nearest completed work stage. Cardiorespiratory, EMG and NIRS variables were calculated as the mean of the final 30 s of the corresponding stage, while neuromuscular variables were obtained during the inter‐stage interval immediately following completion of that stage. For the 100% time point corresponding to task failure, cycling variables were calculated from the final 30 s preceding task failure, whereas neuromuscular variables were assessed immediately at task failure.

In order to account for intra‐individual differences in time to exhaustion and number of stages completed between conditions, a secondary individual ‘isotime’ analysis was also conducted. Specifically, for each participant, neuromuscular, gas exchange and NIRS variables were extracted at the time corresponding to the shorter TTF between conditions, and paired comparisons between SLC and DLC were performed. For example, if a participant reached task failure during stage 8 in DLC and during stage 7 in SLC, stage 6 represented the last stage completed in both conditions and was therefore used as the final common stage for isotime comparisons. Subsequently, for each participant, responses were expressed at 25%, 50%, 75% and 100% of the intra‐individual last common stage number (i.e., relative to the total number of stages completed in both conditions). This secondary analysis was conducted to ensure that between‐condition differences were not attributable to differences in total exercise duration or stage progression, and to permit comparisons at approximately matched mass‐specific (per‐active‐limb) external workloads, given that SLC work rates were set at half those of DLC.

#### 2.7.2 Force and electromyography

Outcome measures from the force measurements included MVC, quadriceps potentiated twitch force (*Q*_tw, pot_) and VA, while the outcome variable for EMG was EMG root‐mean‐squared (EMG_RMS_) measured during cycling. For pre‐exercise measurements, the maximum of the five MVCs was recorded, while the mean of the three *Q*_tw, pot_ and VA values was used for analysis. Values for the same variables from the single assessment taking place between each stage and at task failure were recorded. For the assessment of VA, the following equation was used (Merton, 1954):

$$\%\mathrm{VA}=\left(\;\frac{1-\mathrm{SIT}}{Q_{\mathrm{tw},\mathrm{pot}}}\;\right)\times 100$$

where SIT is superimposed twitch and *Q*_tw,pot_ is potentiated twitch force. Measurements of EMG during cycling were normalised to maximum EMG_RMS_. Maximum EMG_RMS_ was calculated over a 0.2 s epoch around the peak torque of each of the five baseline MVCs, and subsequently averaged. For measurement of EMG during cycling, the RMS signal (0.2 s smoothing window) was visually inspected from the raw data files to determine an appropriate threshold for EMG onset and offset during pedal rotations. Once an appropriate threshold was determined, a macro was subsequently created using LabChart software (ADInstruments) with the appropriate onset and offsets defined, and the EMG_RMS_ was measured between these points.

#### 2.7.3 NIRS

Outcome measures for NIRS included O_2_Hb, HHb, THb and TSI, with the former three variables taken as the average of the three optodes, and TSI determined as O_2_Hb/(O_2_Hb + HHb). The O_2_Hb, HHb and THb signals were set to zero at the onset of the physiological calibration and again at the onset of stage 1 of cycling following the 5 min resting period to standardise the signals prior to normalisation. This approach is consistent with continuous‐wave NIRS, which provides relative rather than absolute measures of chromophore concentration, with subsequent changes expressed relative to the minimum and maximum values obtained during the physiological calibration. For THb the change relative to the beginning of stage 1 was recorded. Table 1 displays the minimum, maximum and delta values for O_2_Hb, HHb and TSI derived from the physiological calibration prior to the SLC and DLC visits, with no differences found between these variables (*P* ≥ 0.374).

**TABLE 1:** Minimum, maximum and delta values derived from the physiological calibration for oxygenated haemoglobin (O_2_Hb), deoxygenated haemoglobin (HHb) and tissue saturation index (TSI).

	SLC	DLC	*P*
O_2_Hb			
Minimum	−23.56 ± 7.39	−22.77 ± 10.05	0.722
Maximum	12.97 ± 4.1	11.87 ± 5.3	0.374
Delta	36.54 ± 10.16	34.63 ± 13.06	0.436
HHb			
Minimum	−3.32 ± 1.94	−3.40 ± 2.88	0.931
Maximum	13.87 ± 8.36	14.30 ± 6.59	0.685
Delta	17.19 ± 9.18	17.70 ± 7.95	0.675
TSI			
Minimum	33.44 ± 5.39	35.27 ± 8.32	0.456
Maximum	67.63 ± 3.37	68.15 ± 2.61	0.538
Delta	34.18 ± 5.00	32.88 ± 8.36	0.503

*P*‐values obtained by paired *t*‐test.

### 2.8 Statistical analysis

Jamovi statistical software (Jamovi, v. 1.0, 2019, the jamovi project; retrieved from https://www.jamovi.org) was used for all statistical analyses. All data are presented as mean ± SD, with error bars in figures representing SD. Statistical significance was set at an α of 0.05. Normality of the data was assessed by the Shapiro–Wilk test, with no data requiring transformation. Assumptions of sphericity were explored and controlled for all variables with the Greenhouse–Geisser adjustment, where necessary. Time to task failure was analysed using Student's paired samples *t*‐test. A two‐way repeated measures ANOVA (modality × time) was used to assess changes in force, EMG, gas exchange, ventilation, HR and NIRS variables over time during DLC and SLC. This analysis was performed for both the percentage task failure and ‘individual isotime’ (i.e., percentage within‐individual last common stage) data, with the latter reported in Appendeix 1. For neuromuscular variables, comparisons were made relative to pre‐exercise baseline measurements obtained prior to the onset of cycling. For cardiorespiratory, EMG and NIRS variables, comparisons were made relative to the first exercise stage (stage 1), with values derived from the final 30 s of that stage. In the event a significant interaction, Tukey's *post hoc* analysis was performed to locate where differences lay. Partial eta squared (η_p_^2^) was calculated to estimate effect sizes, with values representing small (η_p_^2^ = 0.10), medium (η_p_^2^ = 0.25) and large (η_p_^2^ = 0.40) effects. Cohen's *d* effect size was calculated for focused between‐modality comparisons, and were interpreted as small (≥0.2), moderate (≥0.5) and large (≥0.8).

## 3 RESULTS

### 3.1 Time to task failure and neuromuscular fatigability

**FIGURE 3:**
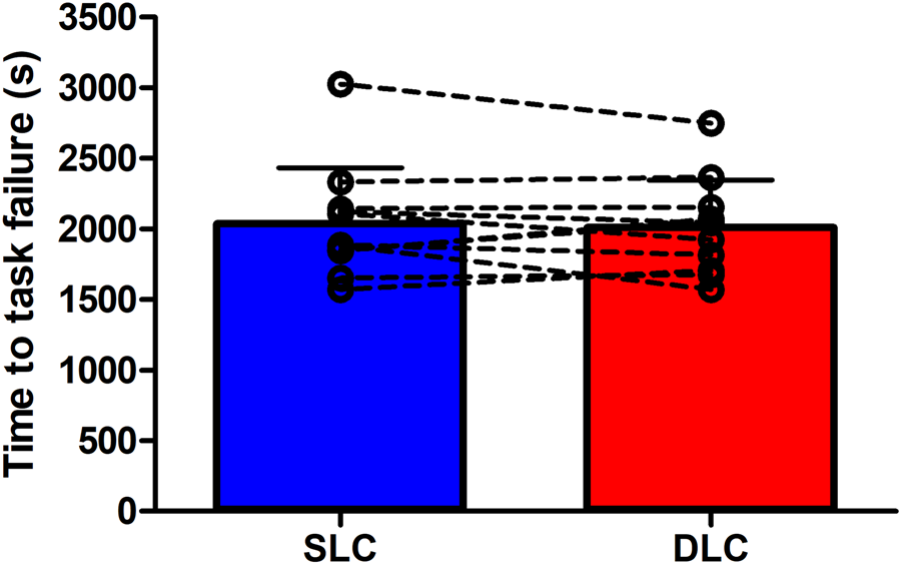
Time to task failure during incremental single leg cycling (SLC) and double leg cycling (DLC) in older adults (*n *= 11). Data are means ± SD; dashed lines show individual data.

Time to task failure (TF) during SLC and DLC is displayed in Figure 3. A paired samples *t*‐test revealed no difference in time to TF between SLC and DLC (*P *= 0.616, *d* = 0.156). Peak power output during the incremental test was 170 ± 34 W for DLC and 87 ± 23 W for SLC. Absolute power outputs associated with stage 1, 25%, 50% and 75% TF for DLC were 16 ± 0, 35 ± 6, 74 ± 11 and 120 ± 34 W, respectively. For SLC, the power outputs associated with the same time points were 8 ± 0, 17 ± 3, 35 ± 4 and 54 ± 15 W, respectively.

**FIGURE 4:**
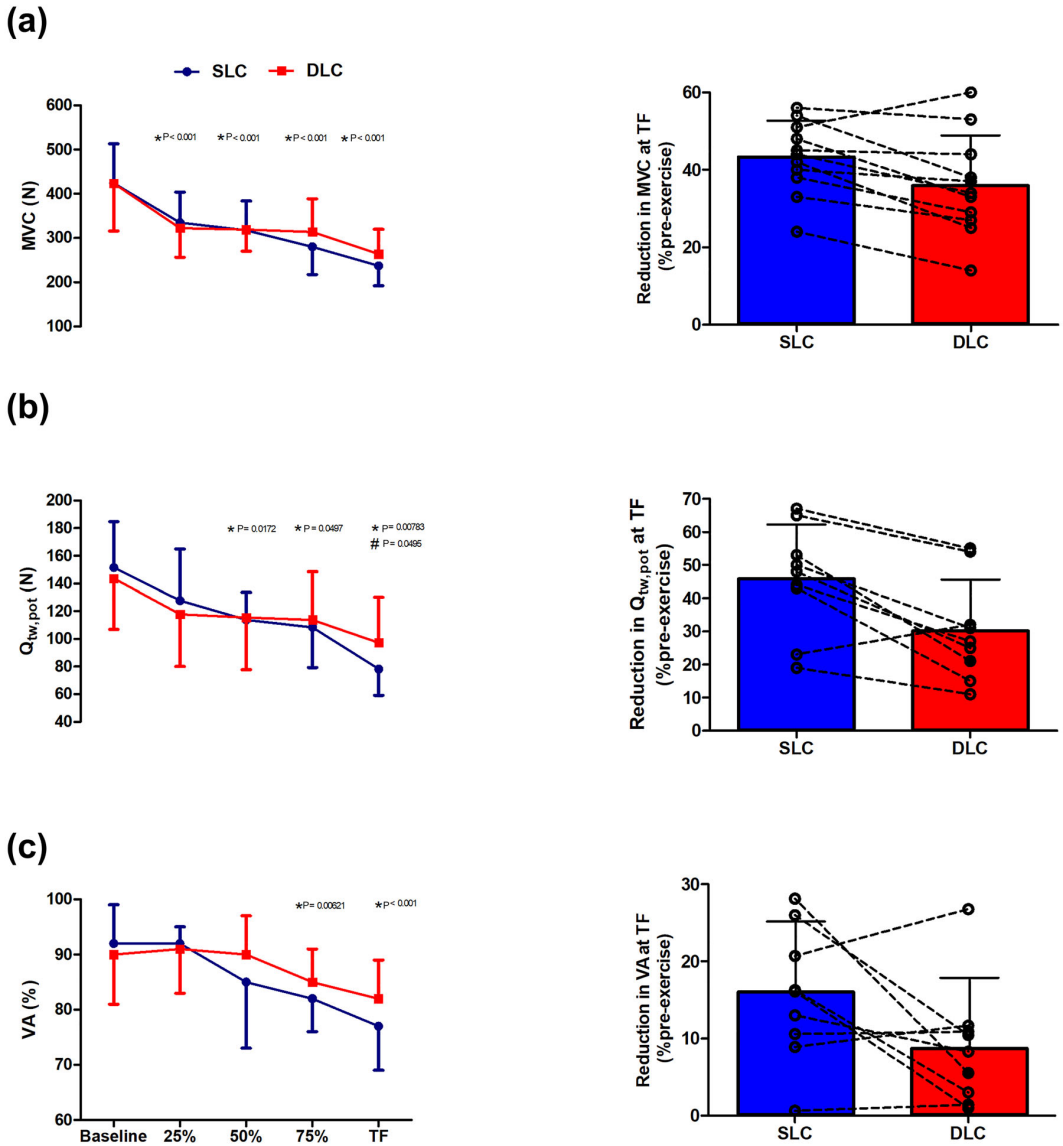
Knee extensor maximal voluntary contraction (MVC, a), quadriceps potentiated twitch (*Q*_tw,pot_, b) and voluntary activation (VA, c) measured during incremental single leg cycling (SLC) and double leg cycling (DLC) in older adults (*n *= 11). Data are means ± SD. *P‐value for comparison vs. baseline (main effect of time); #*P‐*value for between‐modality comparison (time × modality interaction).

For MVC, a main effect of time was found (*F*_1.6,15.6_ = 41.6, *P *< 0.001, η_p_^2^ = 0.806), with MVC lower than baseline at all time points (all *P *< 0.001; Figure 4a; Table 2). No modality (*F*_1,10_ = 1.6, *P *= 0.238, η_p_^2^ = 0.136) or modality × time interaction (*F*_2.3,23.1_ = 1.6, *P *= 0.219, η_p_^2^ = 0.139) was found.

**TABLE 2:** *P*‐values for *post hoc* comparisons following a main effect of time for maximal voluntary contraction (MVC), quadriceps potentiated twitch force (*Q*_tw,pot_) and voluntary activation (VA).

Comparison		MVC	*Q*_tw,pot_	VA
Baseline	25% TF	**<0.001**	0.0849	0.998
	50% TF	**<0.001**	**0.0172**	0.269
	75% TF	**<0.001**	**0.0497**	**0.00621**
	TF	**<0.001**	**0.00783**	**<0.001**
25% TF	50% TF	0.583	0.145	0.0859
	75% TF	**0.0209**	0.215	**0.00291**
	TF	**<0.001**	**0.0213**	**0.00124**
50% TF	75% TF	0.0701	0.615	0.201
	TF	**<0.01**	**0.0149**	**0.0340**
75% TF	TF	**0.00577**	**0.00698**	**0.01434**

Significant differences are highlighted in bold. TF, task failure.

For *Q*_tw,pot_, a main effect of time was found (*F*_1.7,10.6_ = 8.5, *P *= 0.00727, η_p_^2^ = 0.588), with *Q*_tw,pot_ lower than baseline at 50% TF, 75% TF and at TF (*P* ≤ 0.0497; Figure 4b; Table 2). A modality × time interaction was also found for *Q*_tw,pot_ (*F*_4,40_ = 2.8, *P *= 0.0461, η_p_^2^ = 0.321). *Post hoc* analysis showed that *Q*_tw,pot_ was lower at TF for SLC than DLC (*P *= 0.0495, *d* = 0.712; Figure 4b).

For VA, a main effect of time was found (*F*_4,40_ = 12.0, *P *< 0.001, η_p_^2^ = 0.349), with VA lower than baseline at 75% TF (*P *= 0.00621) and at TF (*P *< 0.001; Figure 4c; Table 2). No modality effect (*F*_1,10_ = 3.2, *P *= 0.123, η_p_^2^ = 0.349) or modality × time interaction (*F*_4,40_ = 0.78, *P *= 0.547, η_p_^2^ = 0.115) was found.

### 3.2 Electromyography during cycling

**FIGURE 5:**
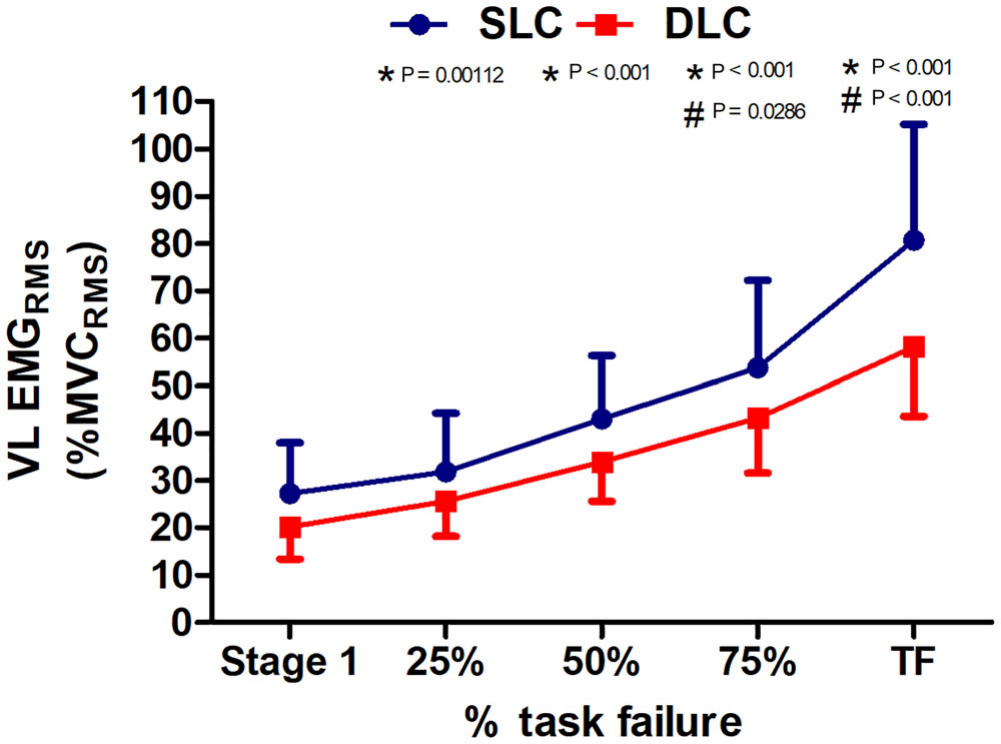
Root‐mean squared electromyography (EMG_RMS_) measured in the vastus lateralis (VL) during incremental single leg cycling (SLC) and double leg cycling (DLC) in older adults (*n *= 11). Data are means ± SD. *P‐value for comparison vs. stage 1 (main effect of time); #*P‐*value for between‐modality comparison (time × modality interaction).

EMG measured in the VL during SLC and DLC is displayed in Figure 5. A main effect of time was found (*F*_1.4,13.8_ = 100.5, *P *< 0.001, η_p_^2^ = 0.909), with EMG higher than stage 1 at all time points (*P *< 0.001; Table 3). A significant modality × time interaction was found (*F*_1.6,15.7_ = 4.7, *P *= 0.0323, η_p_^2^ = 0.320). At 75% TF (*P *= 0.0281, *d* = 0.553) and at TF (*P* = 0.00131, *d* = 1.116), EMG was higher during SLC than DLC.

**TABLE 3:** *P*‐values for *post hoc* comparisons following a main effect of time for electromyography (EMG) measured in the vastus lateralis during cycling.

Comparison		EMG
Stage 1	25% TF	**0.00112**
	50% TF	**<0.001**
	75% TF	**<0.001**
	TF	**<0.001**
25% TF	50% TF	**<0.001**
	75% TF	**<0.001**
	−TF	**<0.001**
50% TF	75% TF	**<0.001**
	TF	**<0.001**
75% TF	TF	**<0.001**

Significant differences are highlighted in bold. TF, task failure.

### 3.3 Gas exchange, ventilation and HR

**FIGURE 6:**
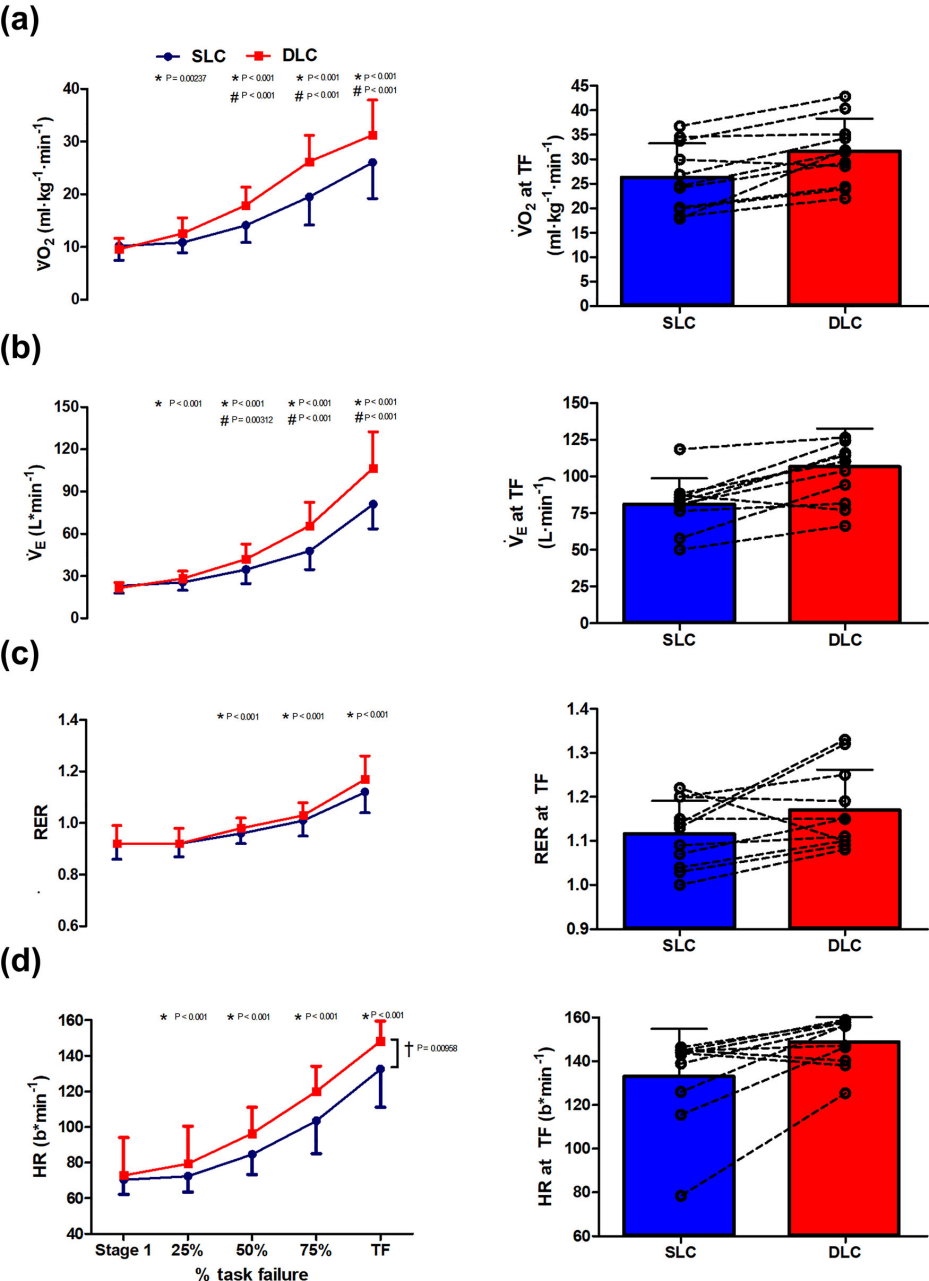
Oxygen consumption (${\dot{V}}_{O_{2}}$, a), ventilation (${\dot{V}}_{E}$, b), respiratory exchange ratio (RER, c), and heart rate (HR, d) measured during incremental single leg cycling (SLC) and double leg cycling (DLC) in older adults (*n *= 11). Data are means ± SD. *P‐value for comparison vs. stage 1 (main effect of time); #*P‐*value for between‐modality comparison (time × modality interaction); †*P‐*value for main effect of modality.

For ${\dot{V}}_{O_{2}}$, a main effect of time (*F*_1.8,17.9_ = 82.3, *P *< 0.001, η_p_^2^ = 0.892) was found, with ${\dot{V}}_{O_{2}}$ higher than stage 1 at all time points (*P* ≤ 0.00237; Table 4). A modality × time interaction was found (*F*_4,40_ = 14.5, *P *< 0.001, η_p_^2^ = 0.592), with ${\dot{V}}_{O_{2}}$ higher during DLC than SLC at 50% and 75% TF, and at TF (all *P* ≤ 0.001, *d* ≥ 1.104; Figure 6a). At TF, ${\dot{V}}_{O_{2}}$ was 1.84 ± 0.38 L min^−1^ (26.04 ± 6.84 mL kg min^−1^) for SLC, and 2.21 ± 0.35 L min^−1^ (31.25 ± 6.63 mL kg min^−1^) for DLC. Thus, ${\dot{V}}_{O_{2}\mathrm{peak}}$ during SLC was 83 ± 12% of that associated with DLC.

**TABLE 4:** *P*‐values for *post hoc* comparisons following a main effect of time for oxygen consumption (${\dot{V}}_{O_{2}}$), minute ventilation (${\dot{V}}_{E}$), respiratory exchange ratio (RER) and heart rate (HR).

Comparison		${\dot{V}}_{O_{2}}$	${\dot{V}}_{E}$	RER	HR
Stage 1	25% TF	**0.00237**	***<*0.001**	0.999	**0.00195**
	50% TF	***<*0.001**	***<*0.001**	**0.0121**	***<*0.001**
	75% TF	***<*0.001**	***<*0.001**	***<*0.001**	***<*0.001**
	TF	***<*0.001**	***<*0.001**	***<*0.001**	***<*0.001**
25% TF	50% TF	***<*0.001**	**0.00218**	***<*0.001**	**0.00171**
	75% TF	***<*0.001**	***<*0.001**	***<*0.001**	***<*0.001**
	TF	***<*0.001**	***<*0.001**	***<*0.001**	***<*0.001**
50% TF	75% TF	***<*0.001**	**0.00193**	**0.0126**	**0.00243**
	−TF	***<*0.001**	***<*0.001**	***<*0.001**	***<*0.001**
75% TF	TF	**0.00988**	***<*0.001**	**0.00732**	***<*0.001**

Significant differences are highlighted in bold. TF, task failure.

For ${\dot{V}}_{E}$, a main effect of time (*F*_2.0,20.1_ = 104.9, *P *< 0.001, η_p_^2^ = 0.913) was found, with ${\dot{V}}_{E}$ higher than stage 1 at all time points (*P *< 0.001; Table 4). A modality × time interaction was found (*F*_1.4,14.2_ = 8.5, *P *= 0.00715, η_p_^2^ = 0.460). *Post hoc* analysis showed that ${\dot{V}}_{E}$ was higher during DLC than SLC at 50% and 75% TF and at TF (*P* ≤ 0.044, *d* ≥ 0.720; Figure 6b).

For respiratory exchange ratio (RER), a main effect of time (*F*_1.8,18.9 _= 42.9, *P *< 0.001, η_p_^2^ = 0.811) was found, with RER higher than stage 1 at 50% TF to TF (*P *< 0.001; Table 4). No modality (*F*_1,10_ = 1.1, *P *= 0.321, η_p_^2^ = 0.098) or a modality × time interaction (*F*_2.1, 21.4_ = 2.12, *P *= 0.142, η_p_^2^ = 0.175) was found for RER (Figure 6c).

For HR, a main effect of time (*F*_1.8,16.1 _= 88.0, *P *< 0.001, η_p_^2^ = 0.907) was found, with HR higher than stage 1 at all time points (*P* ≤ 0.00195; Table 4). A main effect of modality was found (*F*_1,10_ = 10.7, *P *= 0.010, η_p_^2^ = 0.544), with HR higher throughout DLC than SLC, and no modality × time interaction found (*F*_4,40_ = 2.3, *P *= 0.155, η_p_^2^ = 0.200; Figure 6d). At TF, HR was 134 ± 22 and 148 ± 11 bpm for SLC and DLC, respectively, representing 87 ± 14% and 97 ± 7% of age‐predicted maximum.

### 3.4 NIRS

**FIGURE 7:**
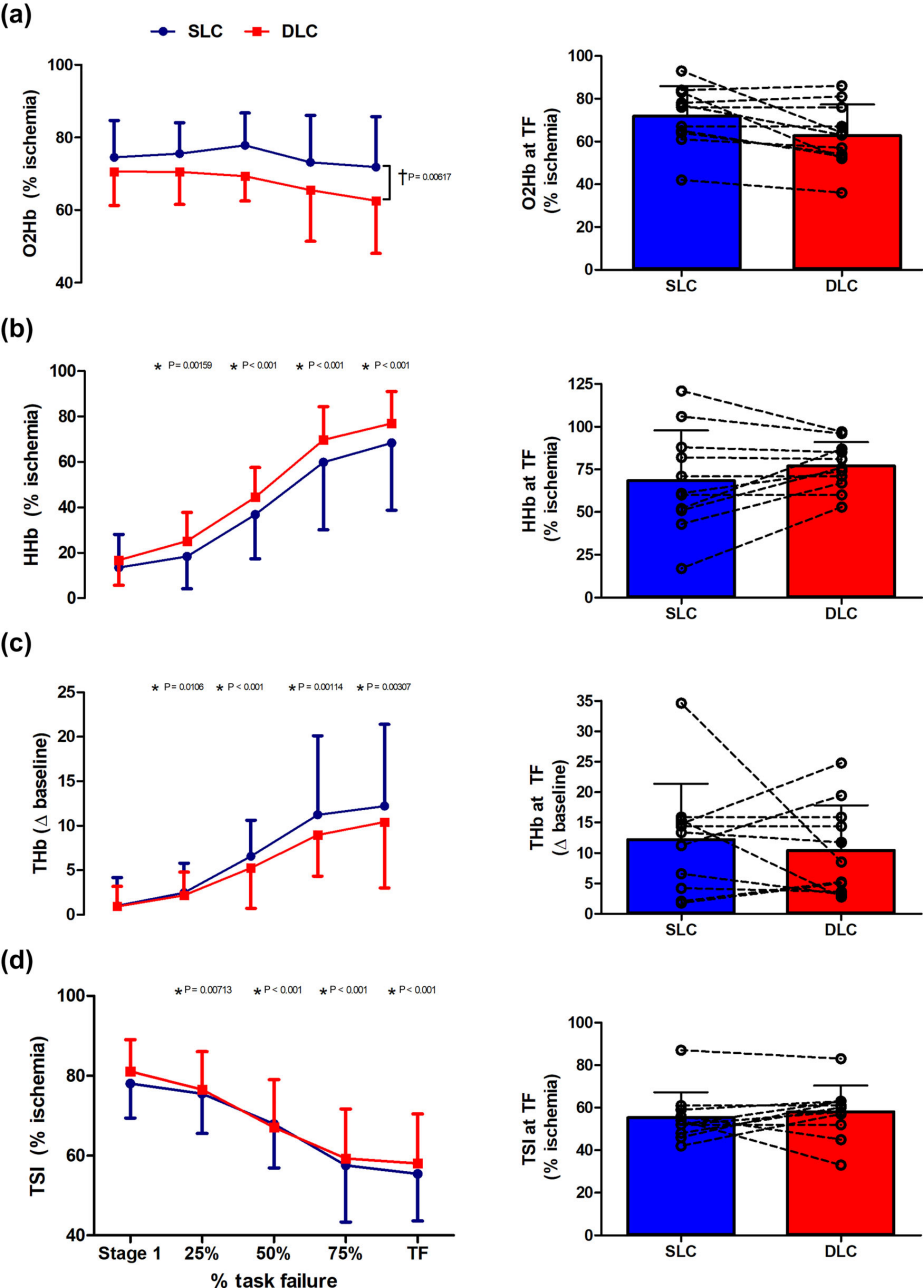
Oxygenated haemoglobin (O_2_Hb, a), deoxygenated haemoglobin (HHb, b), total Haemoglobin (THb, c) and tissue oxygen saturation (TSI, d) measured during incremental single leg cycling (SLC) and double leg cycling (DLC) on the vastus lateralis (VL) in older adults (*n *= 11). Data are means ± SD. *P‐value for comparison vs. stage 1 (main effect of time); †*P‐*value for main effect of modality.

Variables derived from NIRS are displayed in Figure 7. For O_2_Hb, no time (*F*_4,40 =_ 1.13, *P *= 0.356, η_p_^2^ = 0.102) or modality × time interaction was found (*F*_2.0,19.7_ = 2.0, *P *= 0.169, η_p_^2^ = 0.163). However, a main effect of modality was found (*F*_1.0,10.0_ = 12.0, *P *= 0.00617, η_p_^2^ = 0.545), with O_2_Hb higher during SLC than DLC (Figure 7a).

For HHb, a main effect of time was found (*F*_1.3,12.9 =_ 51.6, *P *< 0.001, η_p_^2^ = 0.838) with HHb higher than stage 1 at all time points (*P* ≤ 0.00241; Table 5) and no modality (*F*_1.0,10.0 _= 3.0, *P *= 0.114, η_p_^2^ = 0.230) or modality × time interaction found (*F*_1.8,18.1_ = 0.7, *P *= 0.486, η_p_^2^ = 0.067; Figure 7b).

**TABLE 5:** *P*‐values for *post hoc* comparisons following a main effect of time for deoxygenated haemoglobin (HHb), total haemoglobin (THb) and tissue saturation index (TSI).

Comparison		HHb	THb	TSI
Stage 1	25% TF	**0.00159**	**0.1067**	**0.00723**
	50% TF	***<*0.001**	***<*0.001**	***<*0.001**
	75% TF	***<*0.001**	**0.00114**	***<*0.001**
	TF	***<*0.001**	**0.00307**	***<*0.001**
25% TF	50% TF	***<*0.001**	**0.00119**	***<*0.001**
	75% TF	***<*0.001**	**0.00316**	***<*0.001**
	TF	***<*0.001**	**0.00806**	***<*0.001**
50% TF	75% TF	**0.00946**	0.106	**0.0124**
	TF	**0.00217**	0.0706	**0.00596**
75% TF	TF	**0.0182**	0.465	0.728

Significant differences are highlighted in bold. TF, task failure.

For THb, a main effect of time was found (*F*_4,40 =_ 28.3, *P *< 0.001, η_p_^2^ = 0.739) with THb higher than stage 1 at all time points (*P* ≤ 0.0141; Table 5). No modality (*F*_1.0,10.0 _= 0.8, *P *= 0.406, η_p_^2^ = 0.070) or modality × time interaction was found for THb (*F*_1.2,11.8_ = 0.4, *P *= 0.596, η_p_^2^ = 0.035; Figure 7c).

For TSI, a main effect of time was found (*F*_1.6,16.1 =_ 46.3.3, *P *< 0.001, η_p_^2^ = 0.822), with TSI lower than stage 1 at all time points (*P* ≤ 0.00723; Table 5) and no modality (*F*_1.0,10.0 _= 0.2, *P *= 0.703, η_p_^2^ = 0.015) or modality × time interaction found (*F*_2.4,23.6_ = 0.9, *P *= 0.428, η_p_^2^ = 0.084; Figure 7d).

## 4 DISCUSSION

The present study investigated neuromuscular fatigability and the physiological responses to SLC and DLC in older adults. The results demonstrate a greater decline in *Q*_tw,pot_ during SLC than DLC at task failure, indicative of a greater impairment in contractile function during the former modality. Concurrently, EMG activity was higher at 75% task failure and at task failure during SLC, suggesting a higher muscle activation relative to DLC. This ability to achieve a greater muscle activation and tolerate a more substantial contractile impairment occurred in conjunction with attenuated cardiopulmonary demands, as demonstrated through the lower ${\dot{V}}_{E}$ and HR during SLC than DLC. A further interesting and novel finding from the present study was that O_2_Hb measured using NIRS was higher throughout SLC than DLC, possibly due to enhanced muscle perfusion owing to more favourable conditions for oxygen delivery when competition for blood flow between muscles is reduced. These findings align with previous work demonstrating that small muscle mass exercise elicits greater impairments in contractile function at task failure (Rossman et al., 2012; Weavil et al., 2018; Zhang et al., 2021, 2024). Importantly, the present results extend these observations to older adults and show that the amount of active muscle mass modulates the magnitude of neuromuscular fatigability at task failure, even when exercise modality and mass‐specific work rate are well matched.

### 4.1 Greater impairments in contractile function at task failure following SLC than DLC in older adults

The present study demonstrated that reductions in contractile function were greater at task failure in response to SLC than DLC. Specifically, *Q*_tw,pot_ was reduced by 45.7 ± 18.8% following SLC and 30.0 ± 14.0% following DLC. Notably, the individual isotime analysis indicated that between‐modality differences in *Q*_tw,pot_ were not evident during the shared stages of exercise, and similarly no differences were observed at 25–75% TTF in the %TTF analysis, suggesting that the greater contractile impairment during SLC developed primarily at the limit of tolerance. These findings are similar to previous work investigating neuromuscular fatigability in response to SLC and DLC in young adults. For example, in response to ramp‐incremental cycling, Zhang *et al.* (2021) found that the reduction in *Q*_tw,pot_ was 13% greater at task failure during SLC than DLC. Our findings also align with comparisons of neuromuscular fatigability following small and large muscle mass exercise in older adults. Specifically, Weavil *et al.* (2018) demonstrated that reductions in *Q*_tw,pot_ were ∼15% greater at task failure following dynamic single‐leg knee extension compared with DLC. However, the present study compared neuromuscular fatigability following the same exercise modality between small and large muscle mass exercise (i.e., cycling), thereby isolating the effects of active muscle mass and circumventing the potential confounding influence of differences in exercise modality on neuromuscular fatigability. Thus, the present results confirm that in older adults, when a smaller muscle mass is engaged during exercise, a greater degree of contractile impairment is attained at task failure relative to large muscle mass exercise.

It has been hypothesised that the greater impairment in contractile function commonly observed following small relative to large muscle mass exercise (Zhang et al., 2025) relates to the attenuated central constraints (i.e., neural, cardiac and/or respiratory) when exercising with a smaller active muscle mass. During large muscle mass exercise, limitations in cardiac output and the increased ventilatory demands associated with a higher whole‐body metabolic rate constrain oxygen delivery to the active musculature (Dominelli et al., 2021). These elevated cardiopulmonary demands have also been associated with higher perceptions of effort and dyspnoea during high‐intensity large muscle mass exercise (Abbiss et al., 2011; Zhang et al., 2021), which may contribute to the earlier attainment of task failure (Thomas et al., 2018). During small muscle mass exercise, when these central constraints are attenuated, a greater muscle activation and metabolic stress can be incurred before the perception of fatigue reaches intolerable limits (Thomas et al., 2018). The integrative data from the present study support this suggestion. Specifically, lower cardiopulmonary demands during SLC were confirmed through the HR and ${\dot{V}}_{E}$ data, which, respectively, were 10.8 ± 12.6% and 21.6 ± 17.8% lower at task failure during SLC than DLC. These lower cardiopulmonary demands likely stem from the lower central command, mechanoreflex and metaboreflex when exercising with a smaller muscle mass, thereby attenuating the primary stimuli driving the cardiopulmonary responses to exercise (Fisher et al., 2015; Forster et al., 2012). In turn, higher muscle activation at task failure was supported by EMG_RMS_, which was 80.7 ± 24.5% and 58.2 ± 14.6% MVC_RMS_ at task failure for SLC and DLC, respectively. The greater muscle activity during the latter stages of SLC was likely associated with a higher activation of higher threshold fatigable motor units, and the production of contractile function‐impairing metabolites, such as Pi and H^+^ (Allen et al., 2008), thereby contributing to the exacerbated reductions in *Q*_tw,pot_ at task failure during SLC. Accordingly, these findings indicate that the reduced cardiopulmonary strain during small muscle mass exercise permits greater muscle activation and metabolic stress to accumulate before task failure, thereby exacerbating impairments in contractile function compared with large muscle mass exercise.

In the present study, no significant differences were found in VA between DLC and SLC. Conflicting results exist regarding changes in VA following exercise involving small and large muscle mass exercises. For example, while some studies have reported greater reductions in VA with small muscle mass exercise (Matkowski et al., 2011; Weavil et al., 2018) others have reported no difference (Zhang et al., 2021). Thus, while it has been suggested that large muscle mass exercise might provide greater ‘ensemble’ group III/IV inhibitory afferent feedback, thereby impairing VA (Rossman et al., 2012), the results of the present and prior research do not support this suggestion.

### 4.2 Increased oxygenated haemoglobin during SLC than DLC

An interesting and novel finding from the present study was that, when averaged throughout incremental cycling, O_2_Hb was 8.8 ± 8.0% higher during SLC than DLC. This suggests that the local oxygenation signal within the sampled region of the vastus lateralis was greater during SLC. Given that previous work has shown higher peak blood flow during SLC than DLC (Klausen et al., 1982; LeJemtel et al., 1986), it is tempting to speculate that the higher O_2_Hb might be a result of enhanced blood flow and oxygen delivery during SLC. However, whether higher blood flow was responsible for the greater O_2_Hb during SLC is uncertain. For example, an increase in blood flow would be expected to reduce the reliance on oxygen extraction and in turn result in a lowered HHb, which was not different between conditions in the present study. Furthermore, given that the lower peak blood flow during DLC compared with SLC (Klausen et al., 1982; LeJemtel et al., 1986) is thought to reflect an attenuation of vascular conductance at higher exercise intensities to maintain arterial pressure when cardiac output approaches its limit, one might expect any blood flow‐related differences in O_2_Hb to emerge primarily at the later stages of exercise. However, O_2_Hb was higher on average throughout the entire SLC trial (i.e., main effect of modality), with differences not confined to the later stages of exercise. During these earlier stages at submaximal work rates, differences in mass‐specific blood flow are less likely to explain differences in O_2_Hb. Indeed, previous work has shown that when mass‐specific work rate and ${\dot{V}}_{O_{2}}$ are matched between submaximal single‐ and double‐leg exercise, there are no differences in mass‐specific blood flow (Savard et al., 1989). Thus, the higher O_2_Hb found during SLC in the present study should not be interpreted as direct evidence of greater muscle perfusion during SLC.

### 4.3 Perspectives

Older age is associated with reductions in peripheral vascular and metabolic function, with capillary rarefaction and reductions in mitochondrial function thought to be important contributors to impaired exercise tolerance, as well as various chronic health conditions (Peterson et al., 2012; Ziaaldini et al., 2017). Chronic exercise exposure is a well‐established countermeasure for these impairments, leading to improvements in microvascular and mitochondrial function in older adults (Broskey et al., 2014; Murias et al., 2011). While the optimal exercise stimulus for promoting these improvements is uncertain, the magnitude of intracellular homeostatic perturbations appears to play a central role (Fiorenza et al., 2018, 2020). Given that metabolites associated with substrate level phosphorylation are known to impair excitation–contraction coupling (Allen et al., 2008; Sundberg & Fitts, 2019), the greater impairments in contractile function observed during SLC in the present study indicate an elevated disturbance in the intramuscular biochemical milieu relative to DLC. This is consistent with previous work demonstrating that, during whole‐body cycling, each leg operates at a substantially lower relative intensity (i.e., as a percentage of single‐leg ${\dot{V}}_{O_{2}\mathrm{peak}}$), which may attenuate the local metabolic stimulus experienced by the active musculature (McPhee et al., 2009). In the present study, ${\dot{V}}_{O_{2}\mathrm{peak}}$ during SLC was 83 ± 12% of that achieved during DLC, supporting the notion that each leg operates at a lower relative intensity during whole‐body exercise. Consequently, small muscle mass exercise such as SLC could represent a viable strategy to enhance peripheral adaptations whilst attenuating the cardiac and ventilatory constraints known to impair exercise tolerance in older adults (Molgat‐Seon et al., 2018). Indeed, previous work in young adults has found that SLC is associated with greater mitochondrial adaptations relative to DLC (Abbiss et al., 2011), while studies in healthy (Gordon et al., 2019) and clinical populations (Dolmage & Goldstein, 2008; Tyni‐Lenné et al., 1999) have shown that small muscle mass exercise leads to improvements in tolerance to whole‐body exercise. Accordingly, future research should consider examining and comparing the effects of small and large muscle mass exercise on peripheral vascular and metabolic function, as well as exercise tolerance, in older adults.

### 4.4 Limitations

Several limitations of the present study should be acknowledged. First, the study was conducted exclusively in older adults and did not include a younger comparison group. Consequently, while our findings demonstrate that manipulation of active muscle mass influences neuromuscular fatigability in older adults, the extent to which these responses differ from those observed in younger individuals cannot be directly determined. Previous work has shown that during severe intensity large muscle mass exercise, older adults demonstrate smaller reductions in contractile function at task failure than younger individuals (Krüger et al., 2020), which may reflect lower intramuscular metabolic stress and a greater peripheral reserve due to age‐related cardiopulmonary constraints. Consequently, it remains unclear whether reducing the active muscle mass during exercise might attenuate these central limitations and result in comparable levels of contractile impairment between younger and older adults, which represents an interesting area for future research. Second, the counterweighted single‐leg cycling configuration was used to minimise the mechanical work required during the ascending phase of the crank cycle. While this approach has been validated previously during upright cycling (Elmer et al., 2016), the present study used a recumbent ergometer, which may alter crank mechanics and muscle recruitment patterns. Therefore, although the counterweight was intended to reduce the need for active pull‐up during the pedal cycle, differences in cycling biomechanics between conditions cannot be completely excluded. Third, the interpretation of the NIRS‐derived oxygenation signals should be considered with caution. Continuous‐wave NIRS provides relative changes in chromophore concentrations and does not directly measure muscle blood flow. Moreover, the signals may be influenced by factors such as probe placement, changes in superficial tissue blood flow, and optical pathlength assumptions (Barstow, 2019). Therefore, while the NIRS data provide insight into local muscle oxygenation responses during exercise, definitive conclusions regarding underlying changes in muscle perfusion or oxygen extraction cannot be made.

### 4.5 Conclusions

By manipulating the magnitude of active muscle mass whilst maintaining exercise modality and mass‐specific work‐rate, the present study shows greater muscle activation and impairments in contractile function during incremental SLC compared with DLC in older adults. The greater reductions in contractile function occurred concurrently with reduced overall cardiopulmonary demands. These results suggest that small muscle mass exercise permits a greater tolerable degree of metabolic disruption to be incurred relative to large muscle mass exercise in older adults. These findings contribute to the mechanistic basis for the implementation of small muscle mass exercise to target peripheral adaptations in older adults.

## AUTHOR CONTRIBUTIONS

Callum G. Brownstein conceived and designed the study. Emily Dodd and Callum G. Brownstein performed the experiments, analysed the data, and interpreted the results of experiment. Emily Dodd drafted the manuscript and Callum G. Brownstein edited and revised it. Both authors have read and approved the final version of this manuscript and agree to be accountable for all aspects of the work in ensuring that questions related to the accuracy or integrity of any part of the work are appropriately investigated and resolved. All persons designated as authors qualify for authorship, and all those who qualify for authorship are listed.

## CONFLICT OF INTEREST

None declared.

## FUNDING INFORMATION

None.

## Data Availability

Data available upon request.

## INDIVIDUAL ISOTIME ANALYSIS

### Neuromuscular fatigability

For the individual isotime analysis, the final intra‐individual common stage (i.e., the last completed stage for both SLC and DLC in each participant) was stage 7 ± 2. Consistent with the relative isotime analysis, there were no between‐modality differences in MVC (modality × time interaction *P* ≥ 0.120) or VA (modality × time interaction *P* ≥ 0.176). Similarly, there was no modality × time interaction for *Q*_tw,pot_ (*P *= 0.155), which is consistent with the %TTF analysis showing that reductions in *Q*_tw,pot_ were only greater for SLC than DLC at task failure, a time point not included in the individual isotime analysis.

### Electromyography during cycling

For VL EMG, the individual isotime analysis revealed a main effect of modality, with EMG higher during SLC than DLC (*P* = 0.0212) but no modality × time interaction (*P* = 0.189). In contrast, the %TTF analysis demonstrated higher EMG during SLC at 75% and 100% TTF (see Results). The absence of an interaction in the isotime analysis likely reflects the exclusion of the later stages of the longer trial, where EMG differences were most pronounced as participants approached task failure.

### Gas exchange, ventilation and heart rate

Results from the individual isotime analysis were similar to that of the %TTF, with ${\dot{V}}_{O_{2}}$, HR and ${\dot{V}}_{E}$ higher for DLC than SLC at 50%, 75% and 100% of the last intra‐individual common stage (*P* ≤ 0.0191), while no differences were found for RER (*P *= 0.419).

### Near‐infrared spectroscopy

For O_2_Hb, the individual isotime analysis showed a modality × time interaction (*P *= 0.002), with O_2_Hb higher for SLC than DLC at 50%, 75% and 100% of the final common stage (*P* ≤ 0.002). Results for THb, HHb and TSI were similar to the %TTF analysis, with no condition effects (*P* ≥ 0.0681) and no modality × time interactions (*P* ≥ 0.308).
